# Optical Sensor for Diverse Organic Vapors at ppm Concentration Ranges

**DOI:** 10.3390/s110303267

**Published:** 2011-03-17

**Authors:** J. Christopher Thomas, John E. Trend, Neal A. Rakow, Michael S. Wendland, Richard J. Poirier, Dora M. Paolucci

**Affiliations:** 3M Company, 3M Center, Building 235-2B-87, St. Paul, MN 55144, USA; E-Mails: jtrend@mmm.com (J.E.T.); nrakow@mmm.com (N.A.R.); mswendland@mmm.com (M.S.W.); rjpoirier@mmm.com (R.J.P.); dmpaolucci@mmm.com (D.M.P.)

**Keywords:** chemical sensor, ppm, visual

## Abstract

A broadly responsive optical organic vapor sensor is described that responds to low concentrations of organic vapors without significant interference from water vapor. Responses to several classes of organic vapors are highlighted, and trends within classes are presented. The relationship between molecular properties (vapor pressure, boiling point, polarizability, and refractive index) and sensor response are discussed.

## 1. Introduction

Polymer films have received much attention for their potential use in sensing applications [1,2]. In a previous publication [3], we introduced the design of a sensor using a polymeric detection layer capable of responding to low levels of organic vapors. This sensor construction is valuable for its ability to readily discriminate organic vapors from ambient humidity based on the magnitude of its response. Organic vapors are a chronic exposure concern in home and workplace environments [4], and detecting low levels of organic vapors can lead to reduced exposure [5]. Here, we expand upon the capabilities of the previously introduced sensor by presenting sensor responses to different vapors over a spectrum of organic molecular classes. We examine the magnitude of the response values in terms of shifts of reflectance peak maxima and relate these responses to physical properties of the organic compounds. In particular, the effects of vapor pressure, boiling point, and average molecular polarizability are considered as dominant factors governing sensor response. By studying the current sensor response to a broad range of organic vapors, we have improved our understanding of how the sensor might respond to a known or unknown organic vapor based on the vapor’s physical properties.

## 2. Experimental Section

**Sensor Fabrication**: Generally, sensors were fabricated as previously described [3], with the following modifications: a 4.5% by weight solution of PIM-1 [6,7] in chlorobenzene was used, and the nano-silver metal suspension (DGP-40LT-25C from Advanced Nanoproducts, Korea, 40% by weight silver in methanol) was diluted with an equal mass of isopropanol. After deposition of the silver nanoparticles, the sensor construction was heated in an oven at 125 °C for 5 h to sinter the nanoparticles to form a porous, contiguous mirror.

**Sensor Testing**: Organic vapor test streams, humidity tests, and sensor monitoring were carried out as previously described [3].

**Sensor Response Data**: Sensors were exposed to varying concentrations of a given vapor in a stepwise fashion, starting at low concentration and then increasing concentrations, equilibrating the system as determined by a constant sensor response at each concentration. Sensor responses are reported as **Δ*λ_max_*** as described below, to normalize the response between sensors thereby accommodating the variation in initial maximum peak wavelength between sensors. The ***λ_max_*** of the spectra typically varied between 560 and 580 nm. Sensors were used for a single vapor response concentration series. Reported sensor responses are an average of three to four individual sensor responses. Error bars shown in the figures containing sensor responses indicate the maximum and minimum sensor responses observed across the series. In some instances, data collection was not carried out at certain high vapor concentration levels, particularly for high boiling compounds, due to the difficulties in maintaining a vapor delivery system free of condensed analyte.

## 3. Results and Discussion

### 3.1. Sensor Construction

**Figure 1.**
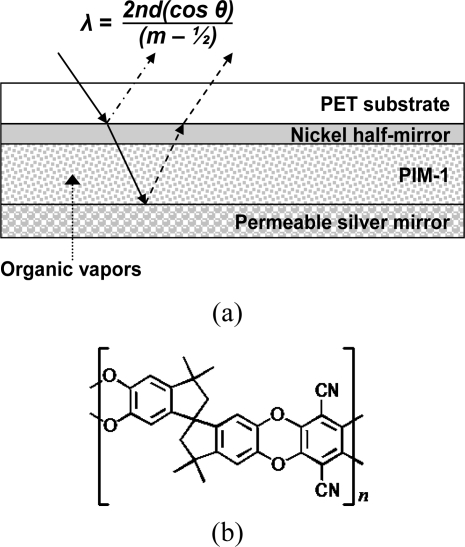
**(a)** Optical indicator construction: a microporous dielectric material is sandwiched between two reflective layers to create a reflective interference filter. The bottom layer is made permeable to organic vapors. Values of ***λ_max_*** for which constructive interference occurs are shown in the equation. **(b)** A thin film of intrinsically-microporous polymer (PIM-1) depicted constitutes the microporous layer in the sensor.

The sensor described herein is depicted in Figure 1. A reflective interference filter is created by positioning a microporous [8] dielectric material (500–650 nm thickness) between two reflective metallic layers. A partial nickel mirror (10 nm thickness) provides partial light reflection while allowing the remaining incident light to travel through the microporous layer. This light traverses the layer a second time upon reflection off the permeable metallic mirror (100–300 nm thickness) to undergo optical interference with the incident light reflected off the partial nickel mirror. Wavelengths for which constructive interference occurs are given by Equation (1):

(1) λmax=2nd cos θ(m−12)

where ***λ_max_*** is the maximum wavelength of a spectral peak, ***n*** is the effective refractive index, ***d*** is the thickness of the microporous dielectric, ***θ*** is the incident angle of light and ***m*** is the integer order number of the reflected peak. Upon sorption of organic vapors by the microporous layer, changes in ***n***, ***d***, or both elicit a shift in ***λ_max_*** and an attendant change in the reflected color of the indicator. This change in ***λ_max_*** can be reported as a wavelength shift, **Δ*λ_max_***, given by **Δ*λ_max_*** = (***λ_max, final_*** − ***λ_max, initial_***). It is worth noting that the sensor also can provide visual indication in addition to spectroscopic responses. As we previously showed, sensor responses on the order of **Δ*λ_max_*** between 15 and 20 nm can provide sufficient optical change in the sensor to be observed visually [3]. This sensor construction is well poised to provide both electronically-monitored spectral shift and visual optical responses to organic vapors.

### 3.2. Sensor Performance

The current sensor response sensitivity depends primarily on the phenomenon of physical adsorption of vapors into micropores and the filling of the micropore void volume over a relatively narrow range of vapor pressures. Secondarily, because PIM-1 is not a highly cross-linked polymeric network, absorption into the polymer bulk can readily occur resulting in a change in physical thickness of the thin film due to swelling. The fraction of the micropore void volume filled at a given vapor pressure and any increase in film thickness caused by swelling combine to produce an increase in the optical path length of the thin film, thereby changing the wavelength condition for constructive reflective interference.

The extent of micropore filling by a given vapor is governed by parameters such as the size of micropores, the surface energy of the pore walls, the partial pressure of the vapor, the polarizability, and hydrodynamic radius of the adsorbed molecule. To be an effective molecular adsorbent of vapors at low concentration, the size of a substantial fraction of the pores within the microporous material must be within a few molecular diameters of the vapor molecule [9]. PIM-1 used herein has been previously characterized as “having a significant proportion of micropores with dimensions in the range of 0.4–0.8 nm [6]”. To be an effective sensor for a breadth of organic vapor classes in air, the sensor needs to be relatively non-selective toward organic molecules, yet highly discriminating against water vapor. We show below that the sensor is highly responsive to a wide range of organic vapors while being generally unresponsive to water vapor.

**Figure 2.**
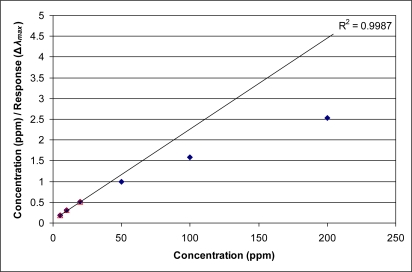
Example of linearization of data for m-xylene, demonstrating the absence of simple Langmuir behavior.

**Figure 3.**
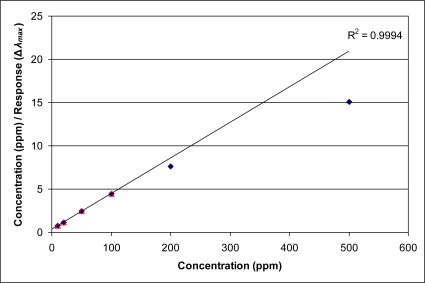
Example of linearization of data for 2,2,4-trimethylpentane, showing closer adherence to simple Langmuir behavior.

The response of the current sensor may best be described using isotherms; wherein, an extensive property (weight gain, volume uptake, and increase in effective index of refraction) is plotted against vapor pressure or concentration (e.g., ppm). Typically, classic isotherms such as Langmuir isotherms and permutations have been used to describe adsorption of gas phase molecules into a microporous solid. The resulting adsorption isotherm curves take the form of square hyperbolas. Such functions can be made linear using reciprocal plots [9]. Figures 2 and 3 shows plots of measured (ppm/**Δ*λ_max_***) for the current thin film sensor *versus* exposure concentration in ppm of m-xylene or 2,2,4-trimethylpentane vapor, respectively. A linear extrapolation applied to the first three data points (5, 10, and 20 ppm) of Figure 2 show that the trend of the response (**Δ*λ_max_***) to *m*-xylene is to deviate from a simple Langmuir isotherm beginning at 50 ppm. In particular, the deviation leads to a larger response (**Δ*λ_max_***) (*i.e.*, larger increase in optical path length) than would be expected from micropore adsorption alone. Figure 3 provides an example of an organic vapor (2,2,4-trimethylpentane) where the response more closely follows simple Langmuir behavior. In this instance, the steric encumbrance of the molecular structure may be resulting in a change in interaction between the pores of the sensor and the organic vapor, leading to more Langmuir-like behavior. The deviation from simple Langmuir behavior for the sensor response is consistently observed to some degree for all of the organic vapors presented here.

Non-Langmuir response behavior in this sensor can be attributed to many factors, including mesoporosity or material swelling. As characterized by Budd *et al*., mesoporosity is not expected to be a significant contributor to the PIM-1 behavior, especially at low partial pressures [7,10]. Polymer swelling by absorption, however, has been shown previously to be a mechanism for sensing vapors at various concentrations [1,2], and for this sensor, swelling may be one mechanism leading to non-Langmuir behavior. Several attributes of the sensors response suggest the deviation from simple Langmuir behavior is due primarily to swelling of PIM-1. First, the deviation from simple Langmuir behavior leads to a higher response than what is expected for just pore filling. Thus, the optical path length of the sensor is increasing greater than that expected for just pore filling. Because the optical path length is defined as the product of the effective refractive index (***n***) and physical thickness (***d***) of the PIM-1 layer, the additional response can be attributed to a change in the physical thickness of the PIM-1 layer. Second, for all organic vapors, simple Langmuir behavior is followed at low concentrations with deviation occurring at higher concentrations of vapor; however, the onset of the deviation occurs at different concentrations for different organic vapors. This behavior is also consistent with a swelling mechanism. Third, a general trend is seen in that the onset and magnitude of the deviation from simple Langmuir behavior is consistent with the solubility of PIM-1 in that solvent. Put another way, organic vapors from solvents in which PIM-1 is more soluble, in general, show a larger deviation from simple Langmuir behavior and at lower concentrations. To conclude, the combination of both microporous adsorption and any other operative mechanisms such as swelling, which we believe to be occurring in this case, result in an enhanced sensor response.

**Figure 4.**
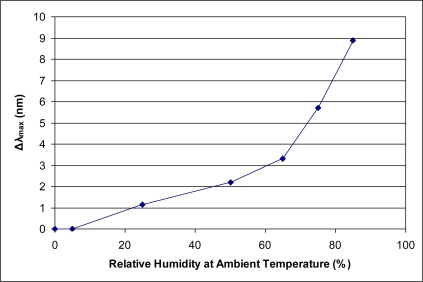
Response of current sensor to increasing relative humidities depicting a Type III isotherm.

A sensor whose function is to detect low levels (*i.e.*, parts per million) of an organic vapor in ambient conditions must be able to discriminate between the organic vapor and various levels of ambient water vapor. The present sensor has been shown previously to provide a relatively low response value (**Δ*λ_max_***) to a high level of relative humidity [3]. At ambient laboratory temperature (typically 22 °C), 85% relative humidity is greater than 22,700 ppm of water vapor. As most organic vapors provide a Type I adsorption isotherm for the sensor response, it is instructive to consider the adsorption isotherm of water vapor, presented in Figure 4. This isotherm is most similar to a Type III isotherm. Type I isotherms are characteristic of a microporous solid interacting favorably with an organic vapor. In comparison, a Type III isotherm is indicative of weak adsorbent-adsorbate interactions [9]. The difference in isotherm types between water vapor and all other organic vapors presented herein suggests that the binding affinity of water into the micropores is significantly less than for organic vapors.

To establish the breadth and capability of the current sensor, performance was evaluated across a range of organic vapors. The organics included in this study are presented in Table 1, along with their vapor pressures, boiling points, and polarizabilities. Organics were chosen from linear alkanes, simple aromatics, ketones, acetates, alcohols, along with selective examples of halogenated alkanes, ethers, and an organonitrile, many of which are common use solvents in laboratory and workplace environments. We present data within these classes of organics from which trends in responses can be observed.

**Figure 5.**
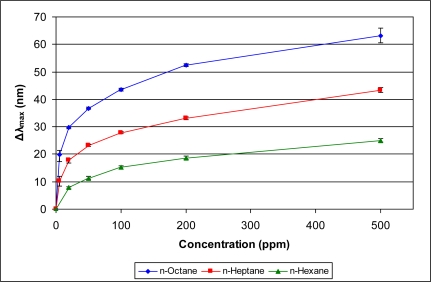
Response of sensor to linear hydrocarbons at various concentrations.

**Figure 6.**
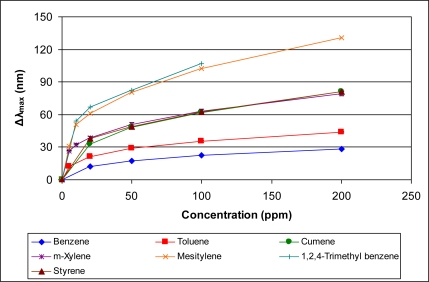
Response of sensor to aromatic hydrocarbons at various concentrations.

**Figure 7.**
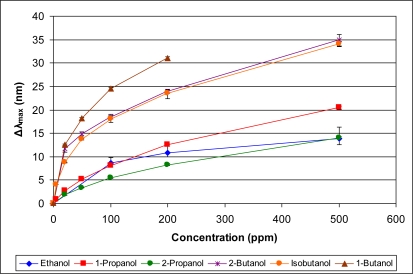
Response of sensor to alcohols at various concentrations.

**Figure 8.**
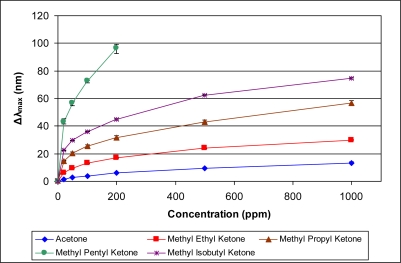
Response of sensor to ketones at various concentrations.

**Figure 9.**
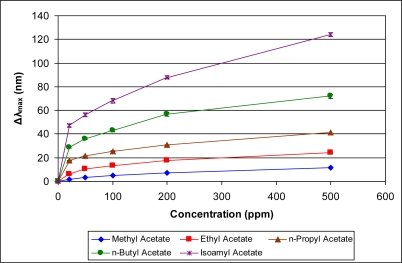
Response of sensor to acetates at various concentrations.

**Figure 10.**
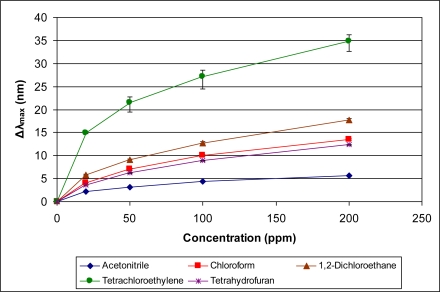
Response of sensor to several organic vapors at various concentrations.

Figures 5–10 present comparative data on sensor responses grouped by simple molecular class (linear hydrocarbons, aromatics, alcohols, ketones, acetates, and other). Data are presented as a function of relative sensor response (**Δ*λ_max_***) *versus* concentration of an organic vapor in ppm. The error bars presented in the response graphs correspond to the range of values obtained from a series of three to four sensors exposed to the same conditions. For uniformly prepared sensors of the present discussion, it is evident that different sensors generally give excellent reproducibility under similar conditions.

The data presented in Figures 5–10 give rise to observable trends in sensor response relative to molecular properties. Linear hydrocarbons represent perhaps the simplest system for comparing organic vapor molecules, as they have minimal electronic or steric functionality. Figure 5 provides the response isotherms for *n*-hexane, *n*-heptane, and *n*-octane. By combining the response data in Figure 5 with the properties in Table 1, an increase in sensor response (**Δ*λ_max_***) correlates inversely in proportion to the vapor pressure and directly in proportion with the boiling point. This trend of increasing response with decreasing vapor pressure is consistent within a given class of molecules for all classes presented in Figures 5–10.

The most notable deviations from the inverse correlation between sensor response and vapor pressure occur when non-linear molecules are directly compared to linear molecules. For example, methyl *n*-propyl ketone (2-pentanone) and methyl isobutyl ketone (4-methyl 2-pentanone) have reasonably different vapor pressures at 20 °C (27 and 16 mm Hg, respectively), but present similar sensor responses (Figure 8). Based on vapor pressure alone, one would expect the sensor to show a larger response for methyl isobutyl ketone. One likely explanation for these results is the ability of the molecules to physically move through the confined spaces of a microporous material. Shape and steric discrimination of molecules to adsorb into microporous materials has been observed previously, as exemplified by the molecular selectivity demonstrated by various zeolites [14] and membranes [15]. Thus, the more hindered methyl isobutyl ketone molecule is more sterically encumbered and may be less able to migrate into the PIM-1 vacancies than the less hindered, linear methyl *n*-propyl ketone molecules. We are continuing to further explore the effects of steric configuration on sensor response. The most significant trend resulting from examining Figures 5–10 is the relationship between sensor response and vapor pressure, which is further modified by additional steric and electronic considerations.

**Figure 11.**
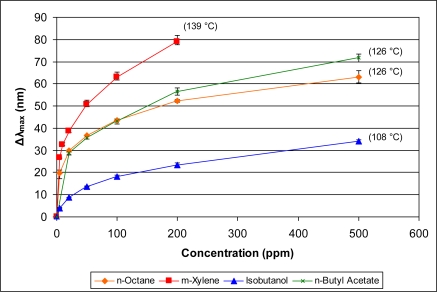
Comparative responses of sensor to organic analytes with similar vapor pressures but different boiling points (noted in parentheses) at various concentrations.

**Figure 12.**
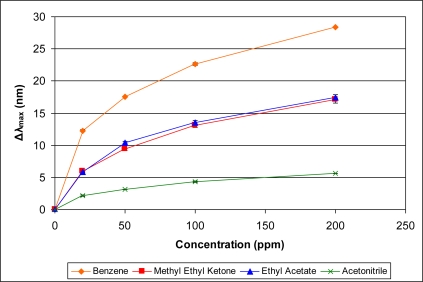
Comparative responses of sensor to organic vapors with similar vapor pressures and similar boiling points at various concentrations.

In addition to examining the responses of the sensor within specific classes of organics, it is also interesting to examine comparative responses across classes. Since the data indicate that vapor pressure is a significant contributor to the sensor response, we compare in Figures 11 and 12 the response of the sensor to diverse vapors with similar vapor pressures. Figure 11 presents the sensor response to *n*-octane, *n*-butyl acetate, isobutanol, and *m*-xylene. The reported vapor pressures at 20 °C of these organics are 10, 10, 9, and 9 mm Hg, respectively (see Table 1). These organics, however, have a range of boiling points (108–139 °C). As can be seen in Figure 11, the sensor response for these four organic vapors is directly proportional to the boiling point.

A comparison of four organics with similar vapor pressures in the range of 73–78 mm Hg is presented in Figure 12. In contrast to the organics presented in Figure 11, the boiling points of the organic vapors in Figure 12 are within a narrow range (77–82 °C). As can be seen from Figure 12, the organic with the highest boiling point, acetonitrile, provides the smallest sensor response. Examining the average molecular polarizability (*α*), though, distinguishes the organics. Acetonitrile has the smallest value of *α* and correspondingly provides the lowest response. Methyl ethyl ketone and ethyl acetate have similar values of *α* (8.25 and 8.87, respectively) and provide very similar responses. Benzene has the highest value of *α* at 10.44 in this group and provides the largest response. Thus, within this group of organics with similar vapor pressures and boiling points, the trend in sensor response correlates directly to the value of *α*, the average molecular polarizability.

It is worth noting that molecular polarizability (*α*) and refractive index (at the sodium D-line and 20 °C, n_D_^20^), which are related through the Lorentz-Lorentz Equation (2):

(2) $$\left(\frac{n_{D}^{20^{2}}-1}{n_{D}^{20^{2}}+2}\right)\frac{M}{\rho }=\frac{4}{3}\pi N_{0}\alpha$$

Both affect the sensor response. With respect to the current sensor and its optical nature, changes in the relative index of refraction are relevant to the magnitude of the sensor response. For a constant volume fraction replacement of air by an organic vapor in the micropores of the sensor material, an organic with a higher refractive index should provide a greater response. If equal numbers of molecules with similar vapor pressures and boiling points are considered, it is anticipated that the average molecular polarizability will affect the interaction between the micropore surface (PIM-1) and the organic molecules. In the current sensor it appears that a larger value of *α* correlates well with greater sensor response. Thus, there may be an additive relationship between molecular polarizability and refractive index that leads to increased sensor response.

It is reasonable to consider whether a relationship is exhibited between the sensor response and other molecular properties as well. Equation (2) incorporates both molecular weight and density. For the comparison presented in Figure 12, the trend in sensor response does not correlate well with either molecular weight (M) or density (ρ) as presented in Table 2. As alluded to earlier, PIM-1 solubility may also be a relevant property that affects sensor response. Solvents for which the PIM-1 is more soluble tend to show greater deviation from simple Langmuir behavior especially at higher concentrations presumably due to swelling. An estimation of the Hildebrand solubility parameter [17] for PIM-1 would allow one to look for a correlation of sensor response to the Hildebrand solubility parameter of the organic liquids. Further work is underway to better understand the correlation between the response of the sensor and the solubility of PIM-1 in a particular organic. It is evident that there are several factors that can influence the response of the current sensor to a specific organic vapor analyte, and further studies will endeavor to elucidate what affects each of these factors.

Several differences between the current sensor and existing technologies for organic vapor monitoring make this current technology attractive. Commonly employed tools for environmental organic vapor monitoring include detection tubes, photoionization detectors (PID), flame ionization detectors (FID), metal oxide semiconductors (MOS), electrochemical sensors, infrared monitors, and gas chromatographs [18]. Of these tools, the current sensor is most comparable to PID-, FID-, and MOS-based technologies in that they all respond broadly to organic vapors. One advantage that the current sensor presents is its ability to be self-calibrating based on its expected optical response. The comparable technologies require regular use of calibration gases to establish a baseline, whereas the baseline of an optical sensor is determined by its optical thickness. Another advantage of the current sensor is its minimal power requirements. Optical measurements typically consume less power than PID-, FID-, and MOS-based measurements. In addition, an optical measurement is not reliant on the composition of the atmosphere for successful use. In contrast, FID and MOS sensors can be dependent on sufficient oxygen to function. A final advantage is the thin-film form of the current sensor. Thin-film technologies have potential economies of scale that can make them competitive in component production. The thin-film construction also provides the opportunity for unpowered response detection by visual inspection, as described previously [3]. To conclude, the current sensor provides a potential means for direct read organic vapor sensing that can compete with currently employed methods.

## 4. Conclusions

We have presented herein response data for a sensor for organic vapors. The data show that the sensor responds at ppm levels to a wide variety of organic vapors. The observed sensitivity and response may be attributed to both micropore filling and changes in the physical thickness of the PIM-1 layer. A polymer swelling mechanism may impact sensor reversibility and recovery time. Notably, water vapor is distinct in not eliciting substantial responses until high atmospheric concentrations are present. This capability to readily distinguish water from other vapors presents a significant sensing advantage when operating under many typically encountered environments. The data evince the effect of vapor pressure on the sensor response: specifically, organics with lower vapor pressures generally have larger responses for a given concentration. Molecular polarizability also appears to contribute to the sensor response in that a large polarizability value may lead to a greater response due to more favorable interactions with PIM-1. Since polarizability is linked to refractive index through the Lorentz-Lorentz equation, there may be additional additive effects in sensor response resulting from the optical nature of the sensor interrogation. Molecular shape and size may affect the sensor response as well. The ability to draw strong correlations of sensor response to various parameters of both the sensor material and the organic analyte will allow for more thoughtful design of sensor materials to achieve selective organic vapor detection. Future studies will further explore the sensor response to additional organic vapors and mixtures.

## Figures and Tables

**Table 1.** Vapors and their properties used in the current studies.

**Compound**	**Vapor Pressure (mm Hg) [11]^a^**	**Boiling Point (°C) [11]**	**Average Molecular Polarizability *α* (Å^3^) [12]**
Acetone	180	56	6.47
Methyl acetate	173	57	7
Chloroform	160	62	8.53
Tetrahydrofuran	132	66	7.97
*n*-Hexane	124	69	11.94
Methyl ethyl ketone	78	79	8.25
Benzene	75	80	10.44
Ethyl acetate	73	77	8.87
Acetonitrile	73	82	4.44
1,2-Dichloroethane	64	83	8.43
Ethanol	44	78	5.13
*n*-Heptane	40^b^	98	13.81
2-Propanol	33	83	6.98
Methyl propyl ketone	27	102	10.11
*n*-Propyl acetate	25	102	10.72
Toluene	21	111	12.4
Methyl isobutyl ketone	16	117	11.98
1-Propanol	15	97	6.96
Tetrachloroethylene	14	121	12.07
2-Butanol	12	99	8.77
*n*-Octane	10	126	15.6
*n*-Butyl acetate	10	126	12.57
*m*-Xylene	9	139	14.33
Isobutanol	9	108	8.81
Cumene	8	152	16.1
1-Butanol	6	117	8.79
Styrene	5	145	14.5
Isoamyl acetate	4	142	14.48
Methyl pentyl ketone	3	152	-
Mesitylene	2	165	16.25
1,2,4-Trimethylbenzene	1^c^	169	-
Water	17.54 [13]	100	1.45

a Vapor pressures are at 20 °C unless otherwise noted.

b At 22 °C.

c At 13 °C.

**Table 2.** Properties of organics compared in Figure 12.

**Compound**	***α* (Å^3^) [12]**	**n_D_^20^ [16]**	**Molecular Weight [16]**	**ρ (g/cm^3^) @ 25 °C [16]**
Benzene	10.44	1.497	78.12	0.873
Methyl ethyl ketone	8.25	1.378	72.12	0.799
Ethyl acetate	8.87	1.37	88.12	0.895
Acetonitrile	4.44	1.344	41.06	0.782 ^a^

a At 20 °C.
